# Production of Biogenic Nanoparticles for the Reduction of 4-Nitrophenol and Oxidative Laccase-Like Reactions

**DOI:** 10.3389/fmicb.2019.00997

**Published:** 2019-05-07

**Authors:** Michael J. Capeness, Virginia Echavarri-Bravo, Louise E. Horsfall

**Affiliations:** ^1^Institute of Quantitative Biology, Biochemistry and Biotechnology, School of Biological Sciences, The University of Edinburgh, Edinburgh, United Kingdom; ^2^Centre for Science at Extreme Conditions, School of Biological Sciences, The University of Edinburgh, Edinburgh, United Kingdom

**Keywords:** biogenic nanoparticles, *Desulfovibrio*, *Morganella*, platinum, palladium, silver, laccase

## Abstract

Biogenic nanoparticles present a wide range of possibilities for use in industrial applications, their production is greener, they can be manufactured using impure feedstocks, and often have different catalytic abilities compared to their chemically made analogs. Nanoparticles of Ag, Pd, Pt, and the bi-elemental PdPt were produced by *Morganella psychrotolerans* and *Desulfovibrio alaskensis* and were shown to be able to reduce 4-nitrophenol, an industrial and toxic pollutant. Nanoparticles were recovered post-reaction and then reused, thus demonstrating continued activity. Biogenic PdNPs were shown to have enhanced specificity in a wide pH activity range in the oxidation of the three common substrates used 2,2′-azino-bis(3-ethylbenzothiazoline-6-sulphonic acid) (ABTS), 2,6-Dimethoxyphenol and (2,6-DMP) and 3,3′,5,5′-Tetramethylbenzidine (TMB) to determine oxidase-like activity. Overall Pd in a nanoparticle form exhibited higher oxidation activity than its ionic counterpart, highlighting the potential of biogenic nanoparticles over the use of ions or chemically made elemental forms.

## Introduction

Nanoparticles (NPs), are particles with a dimension between 1 and 100 nm, which may exhibit different physicochemical properties compared to their bulk-metal equivalent due to their larger surface area to mass ratio. The physicochemical properties of nanoparticles will vary according to their elemental composition, size and surface charge, the latter being dependant on the presence and nature of a capping or coating agent. Developments in nanotechnology exploit the advantageous properties exhibited by nanoparticles. With examples including unique optical properties (Albrecht et al., 2018), antimicrobial abilities (Carvalho et al., 2018), mechanical properties (Gao et al., 2017), and catalytic capabilities (Siddiqi and Husen, 2016; Liu and Liu, 2017). The chemical methods of production of nanoparticles often involves high temperatures, pressures and hazardous chemicals as well as requiring high purity feed stocks (Yu et al., 2009). As such biogenic nanoparticles represent an alternative that has the potential to be greener and a more cost-effective method for the synthesis of nanoparticles (Kharissova et al., 2013).

The term “biogenic” in reference to nanoparticle production covers an array of different methodologies and practices used for the production of nanoparticles, ranging from the use of plant or cell extracts to reduce dissolved metals into nanoparticles, to the use of microorganisms and their innate abilities for the production of nanoparticles. Bacteria represent the most promising area for the study of biogenic nanoparticle production and industrial application due to their ease of growth and genetic manipulation. Especially when coupled with their ability to capture metals from very dilute samples, such as those found as contaminants in the environment or waste streams (Creamer et al., 2006; Nancharaiah et al., 2016).

One such organism, *Desulfovibrio*, is one of the most studied for the production of these nanoparticles, and has been reported to produce nanoparticles of chromium, magnesium, iron, technetium, uranium, nickel, platinum, palladium and zinc (Lovley and Phillips, 1992; Lloyd et al., 1998, 1999; Chardin et al., 2002; Capeness et al., 2015; Gong et al., 2018).

Although *Desulfovibrio* is a popular choice for the synthesis of biogenic nanoparticles, it does not produce nanoparticles of all metals, so new metal-reducing and nanoparticle-making bacterial chassis are becoming more common. In this context, members of the *Morganella* genus have been gaining considerable attention for producing nanoparticles of silver and copper (Parikh et al., 2008; Ramanathan et al., 2011; Pantidos and Horsfall, 2014) as well as for the ability to grow at lower than ambient temperatures and exhibit higher resistance to toxic metals than other Gamma-proteobacteria such as *E. coli*.

Biogenic nanoparticles made by metal-reducing bacteria offer advantages not shared with their chemically made counterparts; (1) bacterial-synthesized nanoparticles can be produced from impure starting materials such as metal-containing waste streams and other feed stocks such as metal contaminated water, (2) as part of a bioremediation process enabling the recovery of valuable metals and adding value to the process, (3) synthesis takes place at ambient conditions and mild temperatures (20–30°C) which reduces operational/manufacturing costs (Vaseghi et al., 2018). Another difference between biogenic and chemically made nanoparticles is the presence of a biological capping agent present on some biogenic nanoparticles, which protects them from oxidation and prevents agglomeration and/or aggregation conferring a higher stability of the nanoparticle (Duran and Seabra, 2012). In terms of their applications, recent studies comparing the activity of biogenic nanoparticles against commercial ones (synthesized chemically) showed that biogenic nanoparticles can be as efficient (Xiong et al., 2018) or even better (Guilger et al., 2017) than the latter.

The aim of the present work was to investigate the properties of AgNPs synthesized by *M. psychrotolerans*, and Pd, Pt, and bimetallic PdPt NPs synthesized by *D. alaskensis* G20 as catalysts for reducing and oxidizing reactions. For comparison a series of colorimetric assays were performed with model compounds. The first assay developed was the reduction of 4-nitrophenol, a biocide and chemical used in the pharmaceutical industry, present in waste streams (Herves et al., 2012). The second set of experiments consisted of investigating the oxidase-like properties of nanoparticles with three different oxidase and peroxidase substrates 2,2′-azino-bis(3-ethylbenzothiazoline-6-sulphonic acid) (ABTS), 2,6-Dimethoxyphenol and (2,6-DMP), or 3,3′,5,5′-Tetramethylbenzidine (TMB) in order to investigate the electrocatalytic properties of the biogenic nanoparticles produced and assess their applications.

## Materials and Methods

### Growth of Strains

*Desulfovibrio alaskensis* G20 was grown on Postgate Medium C using lactate as a carbon source (Postgate et al., 1984). All growth and manipulation was carried out at 30°C in an anaerobic hood fed with 10% H_2_ and 10% CO_2_ in nitrogen. Solutions used with *D. alaskensis* were de-gassed in this chamber prior to use. *Morganella psychrotolerans* U2/3 was grown aerobically in LB with no added salt (LBNS) at 20°C with or without shaking at 200 rpm (Pantidos et al., 2018).

### Production of PdNPs, PtNPs, and PdPtNPs

*Desulfovibrio alaskensis* G20 cells were grown with the previously mentioned conditions to an OD_600_ of 1.0 and in a volume of 500 ml. The cells were then centrifuged at 4000 × *g*, and washed in 10 mM MOPS buffer (pH 7.0) three times all under anaerobic conditions. Na_2_PdCl_4_ and/or PtCl_4_ solutions of 100 mM working suspensions were freshly prepared in ultrapure water (18.2 MΩ cm^−1^) and then added to the cell suspension at a final concentration of 1 mM of each metal. The cell suspension was left for 2 h anaerobically and then centrifuged under anaerobic conditions at 4000 × *g*, the pellet was resuspended in 50% acetone, centrifuged at 13000 × *g*, the supernatant removed and allowed to completely dry. The pellet was then resuspended in ultrapure water. All downstream analyses and manipulations were carried out in aqueous phase solutions prepared in ultrapure water H_2_O.

### Production of AgNPs

*Morganella psychrotolerans* U2/3 was grown aerobically in LBNS at 20°C with shaking at 200 rpm. For the production of AgNPs, a 5 ml overnight culture was used to inoculate a 1 l culture and grown for 24 h, a AgNO_3_ solution was added to a 1 mM final concentration, and the culture incubated for a further 24 h. The suspension was centrifuged at 4000 × g and the cell pellet disposed of. The supernatant was passed through a 0.22 μm filter, then concentrated using cross-flow ultrafiltration in a stirred cell (Amicon), under 2.5 bar pressure of nitrogen through a 5 kDa filter. Once concentrated the filter was washed with 50 ml of H_2_O. The concentrated sample was resuspended off the filter membrane into H_2_O.

All biogenic NPs, once purified, were kept at 4°C, and all subsequent activity reactions were carried out at 20°C. Before use all NPs were sonicated lightly in a water bath for 10 min.

### Transmission Electron Microscopy

Purified NPs and NP/cell suspensions were drop cast on to a 200 mesh carbon-coated copper grid (Agar Scientific), allowed to dry for 5 min, the excess liquid removed, and visualized immediately using a Joel JEM 1400-Plus TEM with an accelerating voltage of 80 kV. Images were captured using a GATAN OneView camera. Image post-processing was carried out using the ImageJ software.

### Energy-Dispersive X-Ray Spectroscopy, Election Energy Loss Spectroscopy, and High-Angle Annular Dark Field Detection

EDS, EELS, and HAADF – STEM (Scanning Transmission Electron Microscopy) samples were prepared as above, though samples were drop cast on holey-carbon nickel grids (Agar Scientific). The samples were analyzed using a JEOL ARM200cF TEM fitted with an ISIS system and viewed at an accelerating voltage of 200 kV. Spectrophotometry measurements were taken using a Gatan 965 Quantum ER spectrometer.

### ICP-OES

Inductively Coupled Plasma Optical Emission Spectrometry was used for ascertaining the amounts of metal ions present in the nanoparticle samples as well as the concentration of the nanoparticles themselves. A sample of each suspension was sonicated for 30 min in a water bath and centrifuged at 20,000 × *g* for 1 h to pellet the NPs, the supernatant (the ionic fraction) was then in added to either 10:14% aqua regia (Pd, Pt, PdPt) or 20% nitric acid (Ag) and heated to 80°C for 8 h. The resulting suspension was diluted in H_2_O to a volume of 3 ml, and subjected to ICP-OES analysis on an Optima 8300 (Perkin Elmer). An unspun sample was also taken (whole fraction), and treated in an identical manner.

### 4-Nitrophenol Assay

All assays were performed in 96 well plates. Suspensions of NPs and their equivalent ions were kept at 0.1 mM final concentration for ease of comparison. In the case of PdPtNPs ICP-OES analysis showed that there was a ratio of 1:1.5 of palladium to platinum, respectively. A solution containing Pd^2+^ and Pt^4+^ ions at this ratio was therefore also used, along with the individual ions (all in H_2_O) for comparative analysis.

The substrate 4-nitrophenol was used at a final concentration of 0.1 mM from a 1 mM stock solution prepared in H_2_O, with reactions taking place in 10 mM NaHB_4_. Plates were incubated at 20°C with constant shaking, with wavelengths of 400 nm being read over the course of 5 min.

For recovery of the NPs used in the above experiments; the entire previous reaction solutions were diluted to 1 ml of H_2_O, centrifuged for 5 min at 20,000 × *g*, washed with 1 volume H_2_O and finally resuspended in 15 μl of H_2_O and then used in a further reduction assay.

### ABTS, 2,6-DMP, and TMB Trials

All reactions were carried out in 96 well plates in a total volume of 200 μl. Readings were taken at 469 nm (2,6-DMP), 652 nm (TMP), and 420 nm (ABTS) over the course of 10 min at 20°C. The buffers used were either 50 mM ammonium acetate buffer (pH 3 or pH 5) or 50 mM HEPES (4-(2-hydroxyethyl)-1-piperazineethanesulfonic acid) buffer (pH 7). NP concentrations of 1 mM were made as previously stated for the 4-nitrophenol assays and used at a final concentrations of 0.1, 0.25, and 0.5 mM. ABTS and 2,6-DMP stocks (20 mM) were prepared in ultrapure water and TMB in 100% EtOH (20 mM), and diluted to a final concentration of 1 mM during the assays.

### Data Analysis

The oxidase activity of PdNPs, PtNPs and bi-metallic PdPtNPs were analyzed by a two-way ANOVA (factors: catalyst concentration and pH). When this analysis was not feasible due to the failure of the normality or equal variances tests (even after data transformation) an alternative test was conducted (e.g., *t*-test).

## Results

### Production of PdPtNPs, PdNPs, PtNPs, and AgNPs

**FIGURE 1:**
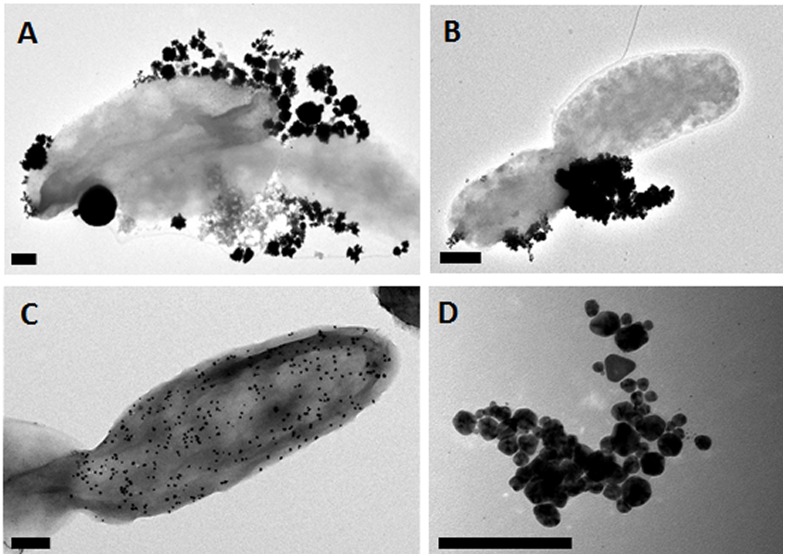
Representative Electron micrographs of nanoparticle samples. **(A)** PdPtNPs, **(B)** PtNPs, and **(C)** PdNPs synthesized by *D. alaskensis* and exhibited on the cell surface. **(D)** AgNPs synthesized by *M. psychrotolerans* free in the cell medium. Scale bar = 200 nm.

**FIGURE 2:**
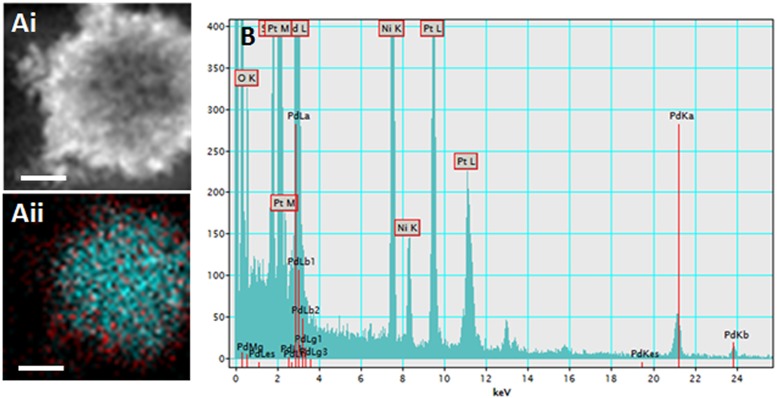
**(Ai)** Electron micrograph of the PdPtNPs (produced after 20 h incubation with *D. alaskensis*) using HAADF **(Aii)** and a composite of the detection for palladium (red) and platinum (cyan). EDS trace **(B)** showing peaks for both palladium and platinum. Showing the elemental composition of the NPs and the distribution of the palladium and platinum metals. Scale bar = 20 nm.

PdPtNP production was shown to be possible by *D. alaskensis* (Figure 1A). PdPtNPs were displayed extracellularly on the cell akin to those of platinum and palladium (Figure 1B,C) and previously reported (Capeness et al., 2015). Unlike the NPs produced by *D. alaskensis*, AgNPs are produced extracellularly by *M. psychrotolerans* and were therefore free within the culture medium (Figure 1D). EELS and EDS showed that the PdPtNPs that were formed were made of both palladium and platinum and both were of an elemental form (Figure 2). Previous work using this method has shown that both the PdNPs and the PtNPs, when synthesized individually, are also made of elemental forms (Capeness et al., 2015). Furthermore, HAADF-STEM and EDS-STEM analysis of the PdPtNPs showed their structure consisted a core of platinum surrounded by palladium (Figure 2Ai,ii). The Pd and Pd (ICP-OES) concentration in the PdPt NPs suspension indicated that the ratio of Pd to Pt was 1:1.5.

### Reduction of 4-Nitrophenol by AgNP, PdNPs, PdPtNPs

**FIGURE 3:**
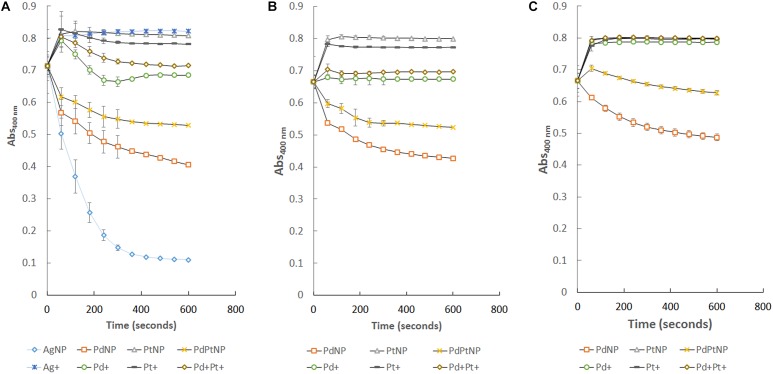
Reduction of 4-nitrophenol over time by the different NPs produced by *M. psychrotolerans* and *D. alaskensis* and by the equivalent ionic forms of those metals. **(A)** Standard reaction, **(B)** re-isolation of those nanoparticles from **(A)** and their use in an equivalent reaction. **(C)** Further re-isolation of nanoparticles from **(B)** and further catalysis of 4-nitrophenol.

The substrate 4-nitrophenol was successfully reduced in the presence of all the nanoparticle types used except PtNPs. AgNPs, PdNPs, and PdPtNPs, reduced the original absorbance to that of 14, 52, and 68% of the starting amount, respectively, over the course of 600 s in comparison to the untreated sample (Figure 3A). The equivalent amounts of dissolved metal ions had little to no effect on this reduction. The PtNPs showed no ability to reduce 4-nitrophenol, so it is hypothesized that even though the PdPtNPs did show a reduction, it is due to the presence of palladium in these NPs. The loss of absorbance at 400 nm corresponds to the reduction of 4-nitrophenol to 4-aminophenol as previously reported (Islam et al., 2018; Nishanthi et al., 2019). However, the amount of reduction measured was greater than that of the Pd content relative to the reduction of 4-nitrophenol by the PdNPs alone. Also unlike the previously reported paper our PtNPs did not affect the reduction of 4-nitrophenol.

Following the successful reduction of 4-nitrophenol by PdNPs and PdPtNPs, recovery of these NPs by means of centrifugation showed that the NPs attached to *D. alaskensis* cells were able to be re-isolated and reused successfully in two further reduction assays (Figure 3B,C). Unlike those of NPs that were attached to cells, it was not possible to re-isolate AgNPs for further catalysis.

### Biogenic NPs Exhibit Oxidase Activity With Laccase Substrates

The oxidase activity of Pd, Pt, and PdPt nanoparticles and their ionic counterparts was pH dependant and increased with the concentration of the metal (Table 1). The results obtained highlight the high selectivity of PdNPs for specific oxidase-substrates depending on the pH. Detailed information covering relevant statistical analysis is available in the Supplementary Material (Supplementary Table 1). A summary of the main factors governing the oxidase activity of biogenic nanoparticles is provided next according to each substrate.

**Table 1:** Oxidase-like activity (nmol min^−1^) measured for different forms of Pd and Pt containing nanoparticles or ions at a concentration of 0.5 mM on the substrates 2,6-DMP, TMB and ABTS.

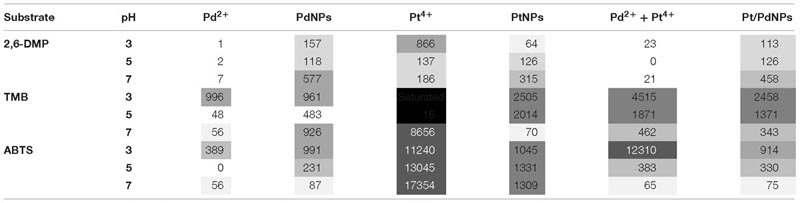

*Oxidase activity (nmol min^−1^) 0–50*
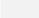
*50–100,*
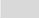
*100–200,*
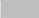
*200–500,*
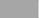
*500–1000,*
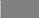
*1000–5000,*
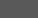
*5000<,*
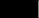
*saturated.*

#### TMB

**FIGURE 4:**
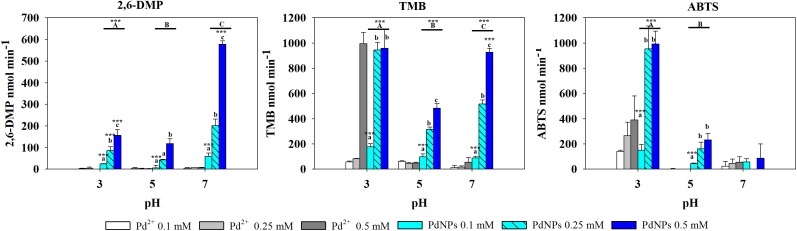
Oxidase-like activity of PdNPs and Pd^2+^ ions at different concentrations of 2,6-DMP, TMB and ABTS across different pH’s, expressed as the mean ± SD. Columns with different letters [capital letters (A,B,C) show differences between pH’s; lower case (a,b,c) show differences between PdNPs concentration] (ANOVA, *p* < 0.05, *n* = 3) ^∗∗∗^ symbolizes statistically significant differences (*p* < 0.05), (*p* < 0.01), and (*p* < 0.001), respectively.

In the presence of TMB, the oxidase-like activity of PdNPs was significantly higher than the activity exhibited by the Pd^2+^ ions except at 0.5 mM and pH 3. The oxidase activity of the PdNPs varied significantly depending on the pH and the concentration tested (two-way ANOVA, *p*-value <0.001) (Figure 4).

**FIGURE 5:**
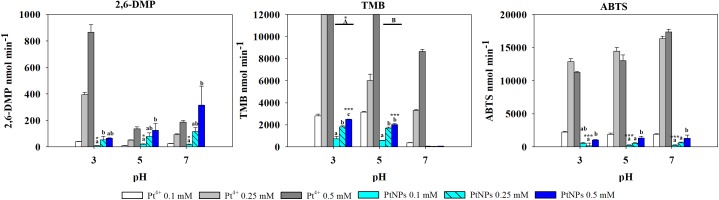
Oxidase-like activity of PtNPs and Pt^4+^ ions at different concentrations on 2,6-DMP, TMB and ABTS across different pH’s, expressed as the mean ± SD. Columns with different letters [capital letters (A,B) show differences between pH’s; lower case (a,b,c) show differences between PtNPs concentration] (ANOVA, *p* < 0.05, *n* = 3) ^∗^ and ^∗∗∗^ symbolizes statistically significant differences (*p* < 0.05), (*p* < 0.01), and (*p* < 0.001), respectively.

The results obtained with Pt showed a different pattern. Ionic Pt^4+^ always exhibited higher oxidase activity than their nanoparticle counterparts, and at concentrations of Pt^4+^ higher than 0.1 mM the reaction with TMB became saturated immediately, thus the absorbance values registered were not adequate (Figure 5).

**FIGURE 6:**
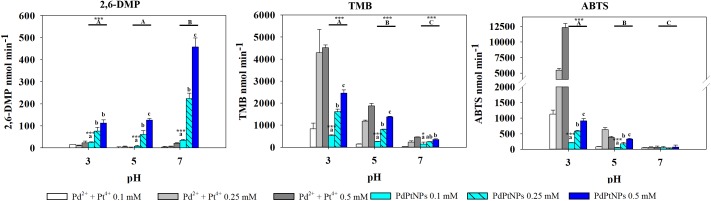
Oxidase-like activity of PdPtNPs and equivalent ions at different concentrations of 2,6-DMP, TMB and ABTS across different pH’s, expressed as the mean ± SD. Columns with different letters [capital letters (A,B,C) show differences between pH’s; lower case (a,b,c) show differences between PdPtNPs concentration] (ANOVA, *p* < 0.05, *n* = 3) ^∗^ and ^∗∗∗^ symbolizes statistically significant differences (*p* < 0.05), (*p* < 0.01), and *p* < 0.001), respectively.

PtNPs and bimetallic PdPtNPs (Figure 6) showed a similar oxidase activity: with both nanoparticle types the oxidation was significantly higher at pH 3 than at pH 5 and pH 7 (ANOVA, *p*-value <0.001). The oxidase activity increased significantly with the concentration of the nanoparticles (PtNPs and PdPtNPs) (*p*-value <0.001). No differences between the PtNPs and the bimetallic NPs were observed at pH 3 and the three concentrations tested (*t*-test, *p*-value >0.05).

#### ABTS

Pt^4+^ ions exhibited the highest oxidase activity compared to the other metallic species tested, and increased significantly at increasing concentrations and pH (two-way ANOVA, *p*-value <0.001). PtNPs showed significantly higher oxidase activity than the PdNPs and bi-metallic PdPtNPs at 0.1 mM (one-way ANOVA, *p*-value = 0.002) and with PdNPs at 0.25 mM (one-way ANOVA, *p*-value = 0.009). However, at increasing concentrations (0.5 mM) no differences were observed between the three nanoparticle types. The Pd ions and PdNPs showed the highest activity at pH 3 although they were was always weaker than Pt-based catalysts. Additionally, the oxidase activity exhibited by the PdNPs was statistically significantly higher than Pd^2+^ (two-way ANOVA, *p*-value <0.001) at 0.25 and 0.5 mM at pH 3.

#### 2,6-DMP

PdNPs showed statistically significant oxidase activity across the three different pH values tested, especially at a concentration of 0.25 mM PdNPs. In addition, PdNPs showed statistically significant higher oxidase activity than the ionic Pd^2+^, especially with 2,6-DMP (Figure 4). The bimetallic nanoparticles showed a similar oxidation pattern to the PdNPs and higher oxidase activity at increasing concentration of nanoparticles, and significantly higher at pH 7. PdNPs exhibited statistically significantly higher activity than PdPtNPs at 0.5 mM. PdNPs also higher oxidation activity than PtNPs, at pH 3 and pH 7 across all the concentrations tested (two-way ANOVA, *p*-value <0.05). The activity of PtNPs was not significantly different across different pH except under two circumstances (pH 3 vs. pH 5 at 0.1 mM PtNPs and between pH 3 and pH 7 at 0.5 mM PtNPs).

Overall Pt^4+^ ions exhibited the highest oxidizing activity with very little selectivity for substrates/pH (Figure 6), being only surpassed in their catalytic action by PdNPs at pH 7 with 2,6 DMP (Figure 4) as a substrate. Interestingly, when Pd^2+^ and Pt^4+^ were present together in ionic form, the oxidase activity decreased significantly (TMB pH 7, ABTS pH 5–7) or was almost suppressed as observed with 2,6-DMP (all three pH tested).

## Discussion

The biogenic metallic nanoparticles synthesized in the present work were able to catalyze both reduction and oxidization reactions with model substrates. Whilst AgNPs synthesized extracellularly by *M. psychrotolerans* exhibited the highest reducing activity in the 4-nitrophenol assays, followed by the platinum-group metal nanoparticles (PdNPs and PdPt NPs), synthesized by *D. alaskensis*. The additional advantage of Pd and Pt based nanoparticles when synthesized by *D. alaskensis* is their recyclability; they can be recovered easily by centrifugation and reused in successive reactions due to being supported on bacterial cells, offering important cost savings, as well as reproducibility.

Oxidizing activity was observed with the platinum-group metal nanoparticles produced by *D. alaskensis*, potentially being useful as catalysts in biological systems due to their activity at pH 5–7 (Tao et al., 2013). Previous studies have shown the peroxidase-like activity of Pt and Pd nanoparticles required the presence of H_2_O_2_ to oxidize a substrate (Li et al., 2015), unlike those presented here. While more recent work has reported that chemically made PdNPs exhibited oxidase-like activity with TMB (Fang et al., 2018). In our work the oxidase-like activity was dependent on the nanoparticle type, the substrate used and the pH, although the type of buffer may also affect the oxidative activity of the nanoparticles (Xue et al., 2012). The ionic counterparts of these nanoparticles also exhibited oxidase activity particularly Pt^4+^ which showed high activity across all the different pH’s and with all the substrates. Conversely, Pd^2+^ generally exhibited lower activity than PdNPs, except with TMB at pH 3. In this assay it was observed that at concentrations of Pd^2+^ between 0.1 and 0.25 mM the oxidase activity was negligible in agreement with previous work (Sun et al., 2017) whereas a dramatic increase of the activity was observed at 0.5 mM. These results indicate that the oxidation of TMB by ionic Pd is not proportional to the concentrations used therefore the oxidative properties of Pd^2+^ deserve further investigation as this has not been reported previously and the underlying mechanisms are yet to be determined.

Some factors, such as pH, will govern others that ultimately affect the oxidative activity of nanoparticles, such as the production of intermediate products (such as H_2_O_2_, OH^•^, and ${\text{O}}_{2}^{•}$) (Cheng et al., 2016; Fang et al., 2018). The pH may also affect the nanoparticles themselves by altering the surface charge (zeta potential) (El Badawy et al., 2010). Changes in the surface charge of nanoparticles may change the electrostatic interaction with the substrate which could ultimately affect their catalytic activity; for instance, TMB exhibits affinity by negatively charged nanoparticles whereas ABTS exhibit higher affinity for positively charged ones (Yu et al., 2009). In oxidation reactions such as peroxidase-like activity it was observed that pH and therefore surface charge was a key determinate of catalytic ability. This has been observed with iron oxide nanoparticles (Yu et al., 2009; Gao et al., 2017) and both cerium oxide (Asati et al., 2009; Cheng et al., 2016) and MnO_2_ nanoparticles (Liu et al., 2012). Alterations in the surface charge due to changes in pH can also affect the agglomeration/aggregation state of nanoparticles. El Badawy et al. (2010) observed that at pH 3 the agglomeration state of silver nanoparticles increased. Our data show that at pH 3, PdNPs did not exhibit any difference in activity between 0.25 and 0.5 mM with TMB and ABTS. One of the hypotheses is that at pH 3, changes in the surface charge of the PdNPs occurred, this together with high concentrations of nanoparticles (0.5 mM) could have led to increased aggregation/agglomeration states, as observed by Baalousha (2009) with iron oxide nanoparticles. Aggregation/agglomeration of nanoparticles may have caused a reduction in the active surface area or nanoparticles mitigating their catalytic activity which would explain the similarities in the oxidase activity observed between 0.25 and 0.5 mM. This is just one of many chemical properties that may influence the catalytic ability of nanoparticles.

Nanoparticles able to catalyze reactions similar to oxidase enzymes have the potential for the development of assays and sensors in various industries. Colorimetric sensors using nanoparticles in the food industry for the detection of sulfites (Qin et al., 2014) as well as their use in immunoassays (ELISA) have already been developed. As biogenic nanoparticles require no enzyme or H_2_O_2_ addition, it opens up their use to a wide range of applications (Asati et al., 2009; Mohamad et al., 2018). Our results showed that the biogenic PdNPs produced by *D. alaskensis* G20 exhibit potential application for developing colorimetric sensors as it showed specificity for substrates depending on the pH; ABTS at low pH (pH 3), 2,6-DMP and neutral (pH 7) and TMB in a biological pH range (pH 5–7). The PtNPs and PdPtNPs did not show advantages over the PdNPs. Current work (Capeness et al., 2019) is focused on enhancing the specificity of *D. alaskensis* G20 for Pd and Pt in order to selectively recover these precious metals from a variety of different sources (water from surface runoff (Moldovan et al., 2001), road dusts and soils (Whiteley and Murray, 2003), as well as leachates of spent catalytic converters from cars (Van Meel et al., 2007).

The nanoparticle-synthesizing capabilities of bacteria, and indeed many other organisms, have been known for decades, and the utility of these particles for an array of different purposes has long been acknowledged. However, the underlying genetics and physiology belonging to these organisms has not been studied. Hidden in the genomes and the physiology of the organism lies the ability for the reprogramming and repurposing of these pathways that make these small highly sought after particles. In recent years, the emergence of synthetic biology and its potential to be a powerful genetic engineering platform has made it possible to convert non-model organisms into novel chasses for the bioremediation of different metals and chemicals into valuable products (Tang et al., 2018). Both *Morganella* and *Desulfovibrio* sp. have desirable characteristics as they are both found in different environmental compartments often containing high levels of metals and as such show heightened resistance. The way in which these resistance mechanisms work and how they can be exploited will allow us to tailor the resultant particles for specific purposes or even unveil entirely new properties unseen before will be key to their use as bioremediation chasses.

Biogenic and in particular bacterially synthesized NPs have a lot of scope for future development. Not only is their production it considered a greener alternative to traditional chemical synthesis, but their production may also solve a bigger problem in the form of metal scarcity. The world requires increasingly greater amounts of metal, and the availability of it is depleting, the metal-containing waste streams from industry (e.g., spent catalytic converters Van Meel et al., 2007) and everyday use can be mined for useful materials, bringing them back in use and helping to create a sustainable circular economy.

## Author Contributions

MC, VE-B, and LH designed the study and analyzed the data, and prepared the manuscript. MC and VE-B performed the experiments.

## Conflict of Interest Statement

The authors declare that the research was conducted in the absence of any commercial or financial relationships that could be construed as a potential conflict of interest.

## Supplementary Material

TABLE S1 Statistical analyses of oxidase experiments.
